# Polarizing trajectories of colonial ideologies: A latent class growth analysis of historical negation and symbolic exclusion

**DOI:** 10.1111/bjso.70116

**Published:** 2026-08-02

**Authors:** Zoe Bertenshaw, Chris G. Sibley, Danny Osborne

**Affiliations:** ^1^ School of Psychology University of Auckland Auckland New Zealand

**Keywords:** colonial ideologies, longitudinal modelling, polarization, political implications, reactionary mobilization

## Abstract

The impassioned advocacy both for and against Indigenous rights in recent years suggests a polarization of public opinion. Accordingly, we theorize increasingly divided endorsement of two ideologies that challenge Indigenous rights to material and symbolic equality—historical negation and symbolic exclusion, respectively (the ‘Dark Duo’). Using latent class growth analysis, we test for distinct classes of change in the Dark Duo over 16 years (2009–2025) among a national probability sample of New Zealand adults (*N* = 43,020). As expected, results reveal a subgroup of *Progressives* (76.84%) with lower levels of historical negation and symbolic exclusion that decreased over time and a smaller subgroup of *Reactionaries* (23.16%) with higher levels of these ideologies that increased over the same period. Demographic and socio‐political variables predicted class membership, and support for political parties varied across classes. Thus, the current study illustrates the presence and implications of ideological polarization towards Indigenous rights.

Driven by both important advances and setbacks, debates over Indigenous rights have intensified globally in recent years. The settler colonial nation of Aotearoa^1^ New Zealand (i.e. the location of the current study) exemplifies these trends. The previous centre‐left government implemented numerous policies designed to increase equity between Pākehā (i.e. New Zealand Europeans—the settler colonizer group) and Indigenous Māori (e.g. legislation for more Indigenous say in the political and health systems; New Zealand Parliament, 2022a, 2022b). Yet, many of these advances were soon reversed (e.g. New Zealand Parliament, 2024a, 2024b) in a reactionary attack on Māori rights under the current right‐wing coalition government, alongside efforts to undermine core protections in Te Tiriti o Waitangi (Te Tiriti), the founding document of European settlement (New Zealand Parliament, 2024c). In response, protests have ignited on both sides of the progressive‐reactionary spectrum, including one of the largest hikoi (i.e. protest march) in the nation's history demanding the government respect Te Tiriti (Corlett, 2024b).

Similar push‐and‐pull dynamics over colonial (in)equality have emerged globally. For example, the United Nations Declaration of the Rights of Indigenous Peoples (UN General Assembly, 2007) stimulated plans and agreements to recognize Indigenous rights across 148 supporting nations (Office of the High Commissioner for Human Rights [OHCHR], n.d.; Te Puni Kōkiri [Ministry of Māori Development], 2020, 2022). Indigenous rights and racial justice protests also gained momentum through the late 2010s and early 2020s (see Ortiz et al., 2022), including unprecedented international support for the Free Palestine movement (Al Jazeera, 2024). These movements include calls to protect and return land (e.g. Standing Rock in the United States, Wet'suwet'en land defenders in Canada, Ihumātao occupation in Aotearoa), increase political representation (e.g. calls for Indigenous voices in parliament in Australia and Canada), and address systematic colonial injustices (e.g. in response to mass school graves in Canada and the celebration of ‘Australia Day’). Conversely, Indigenous rights have been attacked through violent responses to such protests by governments (e.g. in Chile, Ecuador, Peru and the US; American Civil Liberties Union, 2025; Amnesty International, 2023; Human Rights Watch, 2021, 2025) and far‐right groups (e.g. in Melbourne; Wiliams & Morse, 2025) and through political setbacks (e.g. failed initiatives for Indigenous parliamentary voices in Australia and Chile; Parliament of Australia, 2023; Tschorne, 2023). The height of this reactionary shift is exemplified by Israeli forces' violent colonization and genocide of Palestinians, and the support and complicity of international leaders, institutions and allies.

These developments align with broader patterns of political polarization across Western democracies. For example, so‐called ‘woke’ attitudes became increasingly mainstream, and the Black Lives Matter movement saw record international support (BLM; Armed Conflict Location & Event Data Project [ACLED], 2020). Meanwhile, Donald Trump's first presidency spurred an international resurgence of racist and nationalist discourse and policy, accompanied by rising white supremacist protests and attacks (Byman, 2022; Clark, 2020; Southern Poverty Law Center, 2020, 2024). These developments fostered holistic critiques of racial injustice and colonialism and re‐normalized explicit prejudice (Arnold et al., 2026; Crandall et al., 2018), respectively. In sum, reactionary movements tend to follow the progressive gains of the 2010s and stimulate resistance from the left. These cross‐domain dynamics illustrate a broader pattern of political polarization, within which Indigenous rights politics are becoming increasingly divided.

Although democratic politics both shape and reflect public opinion, it is unclear whether the examples of polarization noted above also characterize the general public. This relationship is widely debated for general political ideologies, with scholars arguing that public opinion either mirrors (e.g. Abramowitz & Saunders, 2008) or resists (e.g. Fiorina & Abrams, 2012) political polarization. Political leaders may pursue policies more extreme than voters endorse and without proper consultation—a salient critique of the coalition's anti‐Māori policies in Aotearoa (e.g. Waitangi Tribunal, 2025)—and protests do not reveal the full population's beliefs. Thus, the strong pushes for and against Indigenous rights in contemporary politics signal the possibility of, and need to investigate, polarization in public attitudes towards Indigenous rights.

## COLONIAL IDEOLOGIES AND POLARIZATION

The current tensions surrounding Indigenous rights are situated within a context of longstanding inequities between Indigenous and colonizing groups. Colonialism has enforced societal systems based on Western beliefs, values and practices across colonial contexts, inherently privileging those who align with these norms and marginalizing those who resist (Maldonado‐Torres, 2007; Mignolo & Walsh, 2018; Quijano, 2000). Although Aotearoa is often praised for its expressions of biculturalism (Te Kāhui Tika Tangata, 2022), mainstream systems are still shaped to Western European norms, while sidelining those of Māori (Mikaere, 2011; Milne, 2013; Smith, 2021; Te Hiwi, 2008). Accordingly, policies to promote equity between Pākehā and Māori face considerable debate, obstacles and backlash (Mutu, 2018; Simon, 2022). Combined with the intergenerational consequences of initial colonization processes—such as violence, land alienation and trade exploitation—these contemporary biases undermine Indigenous wellbeing (González et al., 2022). As such, Māori face worse outcomes across social, economic and health domains (Ministry of Social Development [MSD], 2016). This cycle of harm underscores the need to understand how beliefs that uphold these inequities develop over time.

Given the injustice of these past and present colonial harms, ideological responses serve to justify and sustain them—namely, colonial ideologies. At the core is the foundational belief in colonial superiority and Indigenous inferiority, which underpins the colonial project and legitimizes social hierarchies (Fanon, 1967; Quijano, 2000). This belief can be internalized to produce enduring colonial mindsets: Indigenous peoples may develop internalized inferiority (David & Okazaki, 2006; Rivera Pichardo et al., 2021), while colonizers may adopt beliefs that legitimize their privilege and dominance. These broader logics are expressed in specific colonial ideologies that rationalize and perpetuate inequality.

One such ideology is symbolic exclusion. Grounded in the belief of colonial superiority, symbolic exclusion treats Indigenous culture as unfit for modern society and, thus, rejects it from the mainstream national identity (Sibley, 2010). In doing so, symbolic exclusion discredits the Indigenous community's say over the nation and its resources, undermining support for policies that embrace the Indigenous culture and redistribute resources to Indigenous peoples (Bertenshaw, Sibley, & Osborne, 2025). This ideology emerges as a colonial replacement for beliefs that justify racial privilege based on the minority group's lack of belonging and, therefore, rights to resources (i.e. anti‐immigrant beliefs). Given Indigenous communities' *undeniable belonging* to the nation and the insecurity of settlers' own rightful place within the nation (Sibley, 2010), symbolic exclusion denies the Indigenous culture's place in the modern, mainstream national identity while privileging their own culture.

Another ideology that protects colonial hierarchies is historical negation. To avoid colonizers' responsibility for colonial wrongdoings, histories of injustice are often denied, distorted and forgotten (Kidman, 2017; O'Malley & Kidman, 2018; Valentim & Heleno, 2018). These strategies are less effective, however, when injustices are well documented and nationally recognized, as in Aotearoa where Te Tiriti promised equality‐based conditions for European settlement. Although many injustices remain contested and minimized, including the overriding of Māori self‐sovereignty affirmed in Te Tiriti (Te Kāhui Tika Tangata, 2022), these narratives encounter resistance (Mutu, 2019). In this context of *undeniable injustice*, colonizers may instead deny their relevance to contemporary society to avoid responsibility for reparations (i.e. historical negation; Sibley, 2010). In this way, historical negation aims to achieve *historical closure* (e.g. Bilewicz, 2020), while also shirking responsibility for contemporary reparations (Hanke et al., 2013). Accordingly, historical negation predicts longitudinal increases in opposition to policies that redistribute resources to the Indigenous community (Bertenshaw, Sibley, & Osborne, 2025), protecting existing inequities.

Symbolic exclusion and historical negation are united in the Dark Duo model of colonial ideology (Sibley, 2010), which positions the ‘Dark Duo’ as ideologies that justify colonial inequities and protect the privileged position and image of the colonizer group (see also Sibley & Osborne, 2016). The Dark Duo are thus expressions of an enduring colonial mindset tailored to the specific socio‐historical conditions outlined above. Historical negation and symbolic exclusion are also theorized as colonial‐specific outcomes of more general preferences for social hierarchies that emerge from intergroup threat and competition, which are salient under settler colonialism (Satherley & Sibley, 2018; Sibley & Osborne, 2016). Whereas historical negation is linked to right‐wing authoritarianism (RWA; i.e. the tendency to submit to in‐group authorities), symbolic exclusion is underlaid by social dominance orientation (SDO; i.e. the preference for group‐based hierarchy; for longitudinal evidence, see Osborne et al., 2021). Notably, the Dark Duo predict conservative party preferences (Greaves et al., 2014) and correlate with justification of contemporary systems (i.e. system justification; Castro et al., 2022), highlighting their place within the broader ideological architecture that upholds social, racial and colonial hierarchies.

Given that the Dark Duo shape support for Indigenous rights at the ideological and political levels, we explore the polarization of these colonial ideologies to better understand varied perspectives on Indigenous rights. In the wake of Trump's highly controversial first term, polarization has come to the forefront of public discourse and sparked growing research on the topic. Whereas studies often focus on a *state* of polarization (i.e. opposing positions between groups) with cross‐sectional data, longitudinal approaches can assess the *process* of polarization in which groups increasingly diverge on an issue. We are interested in this process to better understand how differences emerge over time.

Most research examining polarization over time focuses on divisions in general political ideologies (i.e. conservatism and liberalism; Pew Research Center, 2014, 2017) and affect towards political parties (e.g. Iyengar et al., 2012, 2019). Some scholars go beyond general left–right divides to explore polarization on specific policies (e.g. immigration), across social classes (Dochow‐Sondershaus & Teney, 2024) or in the general public (Oosterwaal & Torenvlied, 2010). Yet, no research has directly examined the polarization of attitudes towards Indigenous rights over time. Although different (anti‐)egalitarian ideologies may shift together due to shared foundations and political alignments (e.g. conservatism, SDO and RWA), colonial ideologies are shaped by distinct historical and social contexts and, therefore, require direct study.

Although ideologies are conceptualized as relatively stable systems of belief (Jost, 2006), longitudinal analyses reveal gradual mean‐level declines in historical negation and symbolic exclusion among settler colonizers in Aotearoa from 2009 to 2018 (Bertenshaw et al., 2026). These findings illuminate the Dark Duo's malleability over long periods and the average path of Pākehā towards more bicultural mindsets. Yet, they overlook potential heterogeneity in the rates of change in these ideologies across the population. Scholars also identify variation in the levels of each ideology *across* colonizer and Indigenous communities (Sibley & Osborne, 2016), as well as *within* them in Aotearoa (Bertenshaw, Houkamau, et al., 2025) and the United States (Rivera Pichardo et al., 2023). Although these findings signal the possibility of distinct trajectories of change, only one study to date examines varied change in the Dark Duo.

In Aotearoa, Satherley et al. (2020) revealed slight overall polarization in historical negation and support for resource‐based bicultural policies, yet relatively homogenous change in symbolic exclusion and support for symbolic bicultural policies across different party supporters. Although these findings provide insight into ideological changes along voter lines, they leave a gap in our understanding of polarization within the total population and, accordingly, lack insight into the prevalence of different trajectories of change. Furthermore, Satherley and colleagues' analyses included data up to 2017, thereby missing the wave of anti‐Indigenous political sentiment in the 2020s that has stirred concerns of a reactionary movement.

In sum, the literature signals the possibility of polarization in the Dark Duo over time but does not test for it. Tracking distinct ideological change over extended periods is rare, and we have limited insight into whether such change is uniform or varies across subgroups. It is also unclear how these ideologies map onto broader political trends—political movements can influence or reflect public ideology in different ways, from normalizing those attitudes across the community to radicalizing a subgroup or polarizing opposing groups. With the global rise of far‐right, nationalist and racist politics (Byman, 2022; Clark, 2020; Jay et al., 2019), it is important to understand which of these paths the public follows. Likewise, the apparent growth of reactionary political movements requires an assessment of the extent and prevalence of reactionary ideological groups to gauge the potency of this growing threat to democracy.

Information is also needed on the composition of different ideological trajectories. Details on the demographic makeup of different growth paths are needed to identify the groups most at risk of anti‐Indigenous mobilization and advance theory on how social identities shape ideological change. Similarly, the socio‐political composition of progressive and reactionary trajectories offers valuable insight into the mechanisms underlying shifts in colonial ideologies. Finally, examining the party preferences of different subgroups of change would help clarify how ideological trajectories are reflected in political behaviour and increase understanding of the alignment between ideology and party support. Collectively, this information would help inform strategies for reducing anti‐Indigenous mobilization by identifying groups and mechanisms to target. Within the current political climate, these gaps highlight the need to study the distinct ways in which colonial ideologies evolve over time, the socio‐political preferences and demographics associated with certain change, and the connections between different trajectories and political behaviour. The current study addresses these needs.

## THE CURRENT STUDY

One reason for the limited longitudinal research on ideological polarization is the methodological requirements it entails. Given that, ideologies evolve gradually (Page & Shapiro, 2010), assessments over long periods are required, and large, heterogeneous samples are needed to estimate the presence of different change trajectories. Although separate growth models can compare changes across known groups (e.g. liberals vs. conservatives), more complex models are required to identify subgroups *defined by* their ideological trajectories. Fortunately, latent class growth analysis (LCGA) allows us to test for polarization within the population by estimating distinct classes based on their mean levels and rates of change in the focal ideologies. This analytic approach can also estimate the prevalence, predictors and outcomes of distinct trajectories, thereby addressing the theoretical gaps highlighted above.

We employ LCGA to identify the presence and prevalence of different trajectories of change in historical negation and symbolic exclusion among a large, national probability sample of adults in Aotearoa between 2009 and 2025. Several patterns of longitudinal change are plausible. We could identify either a single class reflecting largely homogeneous change, or multiple classes that differ in intercepts but follow parallel, converging or fluctuating trajectories. Against these alternatives, we expect to see a process of ideological polarization over time. Indeed, given the increasingly strong pushes for and against Indigenous rights over the past decade (e.g. Corlett, 2024a, 2024b), we expect to identify at least one class decreasing, and one class increasing, in the Dark Duo over time, producing increasingly opposing ideological positions between 2009 and 2025.

If our analyses reveal multiple classes of change, we will assess age, gender, and ethnicity as demographic correlates of class membership. Younger people tend to be more progressive than older individuals (Cornelis et al., 2009; Villano & Zani, 2007; Zubielevitch et al., 2023), and age predicts higher levels and slower declines in the Dark Duo (Bertenshaw et al., 2026). Similarly, women and gender‐diverse individuals are usually more progressive than men (Albaugh et al., 2025; Dozo, 2015; Huang et al., 2018; Sidanius & Pratto, 1999), and men—relative to women—demonstrate higher levels of the Dark Duo and slower declines in historical negation (Bertenshaw et al., 2026). Lastly, settler colonizers tend to exhibit higher levels of the Dark Duo than Indigenous communities (Sibley & Osborne, 2016). Accordingly, being older, identifying as a man, and being Pākehā should predict membership in the more reactionary group(s).

We will also assess socio‐political correlates of class membership. Given their role in maintaining colonial inequality (Sibley & Osborne, 2016), the Dark Duo correlates positively with anti‐egalitarian orientations including conservatism (Castro et al., 2022; Greaves et al., 2014), system justification (Castro et al., 2022), and SDO and RWA (Osborne et al., 2021; Satherley & Sibley, 2018). Conservatism and SDO also predict slower declines in historical negation, whereas SDO predicts faster declines in symbolic exclusion (Bertenshaw et al., 2026). Thus, those in the more reactionary group(s) should have higher levels of conservatism, system justification, SDO and RWA than those in the progressive group(s).

Finally, groups displaying progressive and reactionary trajectories of change should also have distinct political party preferences. Given National, ACT and New Zealand. First endorse policies that restrict Indigenous rights (see Corlett, 2024a; National Party & ACT Party, 2023; National Party & New Zealand First, 2023), we should see higher support for these right‐wing parties within the class(es) displaying reactionary trajectories of change in the Dark Duo. In contrast, Labour, Greens, and Te Pati Māori promote policies that empower the Indigenous community (Gibbons, 2024). As such, there should be more support for these left‐wing parties within the class(es) displaying progressive trajectories of change in the Dark Duo.

## METHOD

### Data access

Our data are from the New Zealand Attitudes and Values Study (NZAVS). Full copies of the NZAVS data files are held by all members of the NZAVS management team and advisory board. A de‐identified dataset containing the variables analysed here is available upon request from the corresponding author for the purpose of replicating our results. The NZAVS survey and *Mplus* syntax are available on the OSF: https://osf.io/r4edc/?view_only=4aa8c21167494ad9b877bf6722f5c854. The NZAVS was approved by the University of Auckland Human Participants Ethics Committee on 26/05/2021 for a period of 6 years. Participants gave their informed consent before participating, and their responses are anonymized and confidential (for more information, see https://osf.io/75snb/). We report all exclusions in the study. Our hypotheses and syntax were pre‐registered^2^ (see https://osf.io/jmw9e/?view_only=8ca944b647384fe88ef3da48e5cc453a).

### Sampling procedure

Due to space constraints, our focal variables were not assessed in Times 10–13. As such, we analysed data from Times 1 to 9 and 14 to 16 of the NZAVS—a longitudinal national probability study of adults that began in 2009. Sampling occurred on nine occasions. At Time 1 (2009/2010), a random sample of adults from the electoral roll was invited to participate in a projected 20‐year longitudinal panel study, yielding 6518 participants (response rate = 16.6%). Booster samples were pursued to address sample attrition and increase sample diversity, including random samples from the electoral roll (without replacement) at Time 4 (2012/2013), Time 5 (2013/2014), Time 8 (2016/2017), Time 10 (2018/2019), Time 14 (2022/2023) and Time 15 (2023/2024), with some targeting underrepresented communities. Non‐random booster samples were also recruited at Time 3 (2011/2012) from a newspaper website, Time 11 (2019/2020) by asking whether partners of participants would like to join, and Times 11 (2019/2020), 13 (2021/2022) and 14 (2022/2023) through Facebook/Instagram adverts. At Time 15 (2023/2024), a Muslim community booster sample was recruited, largely through community outreach. By Time 16 (2024/2025), the sample included 31,873 participants (31,393 retained from one or more previous waves). In total, 76,889 participants completed at least one of the 16 waves of the study. For the full sampling procedure, see https://osf.io/75snb/wiki/Sample%20Details/.

### Participants

Of these 76,889 participants, we focus on the 43,020 (55.95% of the full sample) who completed four or more waves of the study and responded to our variables of interest (*M*_age_ = 42.79, *SD* = 13.77; 63.1% women, 35.8% men, 1.1% gender‐diverse). Participants identified as New Zealand European (i.e. Pākehā; 80.8%), Māori (12.1%), Pacific peoples (2.4%) and Asian (4.1%), while 0.6% of participants identified as other ethnicities (i.e. Other). Table S1 displays the sample composition across waves (see Supplementary Materials).

### Measures

#### The Dark Duo

Unless noted, items were rated on a 1 (strongly disagree) to 7 (strongly agree) scale. Historical negation was assessed using three items from Sibley et al. (2008): ‘We should all move on as one nation and forget about past differences and conflicts between ethnic groups’, ‘We should not have to pay for the mistakes of our ancestors’ and ‘People who weren't around in previous centuries should not feel accountable for the actions of their ancestors’ (*α* = .80–.86). Symbolic exclusion was measured using three items from Sibley (2010): ‘I think that Māori culture helps to define New Zealand in positive ways’ (reverse‐coded); ‘I reckon Māori culture should stay where it belongs—with Māori. It doesn't concern other NZers’; and ‘New Zealand would be a better place to live if we forgot about trying to promote Māori culture to everyone’ (*α* = .81–.89).

#### Demographic predictors

Age at Time 1 was assessed by asking participants to report their date of birth. A forced‐choice tick box (Male/Female) was used to assess gender from Times 1 to 5, followed by a more inclusive open‐ended format beginning in Time 6. Responses were coded into man, woman and a collective ‘gender‐diverse’ category due to sample size constraints, including participants who identified as gender‐diverse at any time point.^3^ Ethnicity was (non‐priority) coded as New Zealand European (i.e. Pākehā), Māori, Pacific, Asian and Other, which included all other ethnicities due to sample size constraints. Although the small sample size and ethnic variation within the ‘Other’ group complicate the interpretation of this category, we include it to maintain the independence of the focal ethnic groups in the predictors analysis.

#### Socio‐political predictors

Conservatism was assessed by having participants ‘rate how politically liberal versus conservative you see yourself’ on a 1 (extremely liberal) to 7 (extremely conservative) scale. System justification was measured using four^4^ items from Kay and Jost (2003): ‘In general, the New Zealand political system operates as it should’, ‘In general, I find New Zealand society to be fair’, ‘Everyone has a fair shot at wealth and happiness in New Zealand’ and ‘Most of New Zealand's policies serve the greater good’ (*α* = .58–.79). SDO was assessed using six items from Sidanius and Pratto (1999), including ‘Inferior groups should stay in their place’ and ‘We should have increased social equality’ (reverse‐coded; *α* = .69–.79). RWA was measured using six items^5^ from Altemeyer (1996), including ‘It is always better to trust the judgment of the proper authorities in government and religion than to listen to the noisy rabble‐rousers in our society who are trying to create doubt in people's minds’ and ‘Our country will be destroyed someday if we do not smash the perversions eating away at our moral fibre and traditional beliefs’ (*α* = .63–.75). For all four socio‐political predictors, we used scores from participants' first observed measurement.

#### Political party support

We assessed support for the primary political parties in Aotearoa: National Party, Labour Party, ACT,^6^ Green Party, Te Pāti Māori and New Zealand First.^7^ Participants were asked to ‘…rate how strongly you oppose or support each of the following political parties’ on a 1 (strongly oppose) to 7 (strongly support) scale. We used participants' support for each party at the most recent observed assessment.

## RESULTS

### Latent class growth analysis

To assess classes of change in the Dark Duo, we employed LCGA in *Mplus version* 8.11 (Muthén & Muthén, 1998–2024), using FIML to handle missing data (Enders & Bandalos, 2001). LCGA is a person‐centred approach that allows for different trajectories of change to emerge within the sample and, thus, identifies classes (or subgroups) with varying intercepts and/or rates of change in the focal variables (Muthén & Muthén, 2000). Given that the Dark Duo are related, yet distinct, ideologies that mutually reinforce colonial inequities (Sibley, 2010), we include both variables in the model to identify multi‐trajectory classes of historical negation and symbolic exclusion. Additionally, because historical negation and symbolic exclusion demonstrate curvilinear change (Bertenshaw et al., 2026), we estimated linear and quadratic growth for each class.

We estimated models with between one and four classes of growth trajectories in historical negation and symbolic exclusion, balancing model fit with both parsimony and theoretical contributions to identify the best fitting model. Specifically, we examined the Akaike information criterion (AIC), the Bayesian information criterion (BIC) and the sample‐size adjusted BIC (aBIC), wherein decreases signal improvements to model fit and entropy, wherein higher values (i.e. closer to 1.0) indicate more accuracy in the classification of participants into distinct classes (Wickrama et al., 2021). Although the AIC, BIC and aBIC declined with each additional class, the size of the decline plateaued following the addition of a second class (See Table 1). Thus, the largest improvement to model fit emerged from a one‐ to two‐class solution. The two‐class solution also exhibited the highest entropy (0.720) across the estimated models. Corroborating the fit of the two‐class model, Table 2 reveals that the probability a participant belonged to their assigned class ranged from .855 to .936, indicating a small likelihood of misclassification. We thus concluded that the two‐class solution provided the best fit to our data.

**TABLE 1:** Model fit comparison of one to four‐class latent class growth models.

Model	AIC	BIC	aBIC	ΔAIC	ΔBIC	ΔaBIC	Entropy	Class prevalence (%)			
								1	2	3	4
1 Class	1,037,021.941	1,037,117.304	1,037,082.346	—	—	—	—	100	—	—	—
**2 Classes**	**1,020,437.542**	**1,020,593.592**	**1,020,536.388**	**16,584.399**	**16,523.712**	**16,545.958**	**0.720**	**76.84**	**23.16**	—	—
3 Classes	1,013,451.566	1,013,668.302	1,013,588.852	6985.976	6925.290	6947.536	0.567	62.44	18.38	19.18	—
4 Classes	1,007,452.273	1,007,729.694	1,007,627.998	5999.293	5938.608	5960.854	0.612	19.16	8.05	30.49	42.30

*Note*: Values in bold represent the selected solution. *N* = 43,020.

Abbreviations: AIC, Akaike information criterion; aBIC, sample‐size adjusted Bayesian information criterion; BIC, Bayesian information criterion.

**TABLE 2:** Average latent class probabilities for most likely latent class membership (row) by latent class (column).

Class	*N*	%	1	2
1. Progressives (76.84% of the sample)	33,058	76.84	**0.936**	0.064
2. Reactionaries (23.16% of the sample)	9962	23.16	0.145	**0.855**

*Note*: Values in bold represent the average estimated probability of a ‘correct’ classification.

### Classes trajectories

**FIGURE 1:**
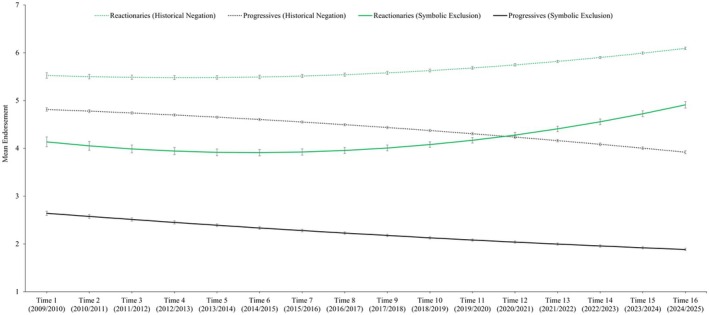
Two‐class latent class growth model of historical negation and symbolic exclusion from 2009 to 2025. Error bars represent 95% confidence intervals. *N* = 43,020.

Table 3 displays the estimates for historical negation and symbolic exclusion in our two classes; Figure 1 plots the estimated mean levels of the Dark Duo for each class from Time 1 (2009/2010) to Time 16 (2024/2025). Most participants (76.8%) belonged to a class with lower levels of historical negation and symbolic exclusion that consistently declined over time. Accordingly, we label this class the *Progressives*. Indeed, this subgroup demonstrated lower—albeit still substantial—levels of historical negation (*i* = 4.49, *SE* = 0.01, *p* < .001) and linear (*s* = −0.58, *SE* = 0.02, *p* < .001) and curvilinear (*q* = −0.18, *SE* = 0.02, *p* < .001) decreases in historical negation that accelerated over time. The *Progressives* also displayed low levels of symbolic exclusion (*i* = 2.23, *SE* = 0.01, *p* < .001), with linear decreases (*s* = −0.51, *SE* = 0.01, *p* < .001) and curvilinear increases (*q* = 0.11, *SE* = 0.02, *p* < .001), indicating that these declines decelerated over time.

**TABLE 3:** Parameter estimates for the two‐class latent growth model of historical negation and symbolic exclusion.

Class		Historical negation					Symbolic exclusion				
		Estimate	*SE*	95% CI		*p*‐value	Estimate	*SE*	95% CI		*p*‐value
				LB	UB				LB	UB	
Progressives (76.84% of the sample)	*i*	4.49	0.01	4.471	4.516	<.001	2.23	0.01	2.200	2.252	<.001
	*s*	−0.58	0.02	−0.607	−0.549	<.001	−0.51	0.01	−0.542	−0.487	<.001
	*q*	−0.18	0.02	−0.218	−0.136	<.001	0.11	0.02	0.068	0.148	<.001
Reactionaries (23.16% of the sample)	*i*	5.54	0.02	5.509	5.574	<.001	3.95	0.03	3.892	4.016	<.001
	*s*	0.33	0.02	0.295	0.373	<.001	0.42	0.03	0.356	0.484	<.001
	*q*	0.44	0.03	0.380	0.504	<.001	0.97	0.05	0.867	1.068	<.001

*Note*: Variances are constrained to equality across latent classes. *N* = 43,020.

Abbreviations: 95% CI, confidence interval; *i*, intercept; LB, lower bound; *q*, quadratic slope; *s*, linear slope; UB, upper bound.

The remaining 23.2% of the sample belonged to a class with higher levels of historical negation and symbolic exclusion that increased over time. Given that they diverged notably from the declines observed among *Progressives*, we label this subgroup the *Reactionaries. Reactionaries* displayed higher levels of historical negation (*i* = 5.54, *SE* = 0.02, *p* < .001), and overall linear (*s* = 0.33, *SE* = 0.02, *p* < .001) and curvilinear (*q* = 0.44, *SE* = 0.03, *p* < .001) increases in historical negation between 2009 and 2025. Thus, the increase accelerated over time. Likewise, they demonstrated higher levels of symbolic exclusion (*i* = 3.95, *SE* = 0.03, *p* < .001) and overall linear (*s* = 0.42, *SE* = 0.03, *p* < .001) and curvilinear (*q* = 0.97, *SE* = 0.05, *p* < .001) increases in symbolic exclusion over time, signalling an accelerating incline in this ideology.

### Predictors of class membership

To understand the demographic and socio‐political makeup of these subgroups, we assessed the relative odds of class membership based on gender, age, ethnicity, conservatism, system justification, SDO and RWA using three‐step multinomial logistic regression (see Table 4). These analyses allow us to estimate the unique influence of the predictor variables on profile membership without impacting the estimation of classes (Asparouhov & Muthén, 2014).

**TABLE 4:** Multinomial logistic regression predicting the likelihood of belonging to the reactionaries relative to the progressives class.

	Reactionaries (relative to progressives)							
	*b*	SE	95% CI		OR	95% CI		*p*
			LB	UB		LB	UB	
Gender^a^								
Woman	−0.58	0.04	−0.653	−0.509	0.56	0.520	0.601	<.001
Gender diverse	−0.50	0.22	−0.919	−0.076	0.61	0.399	0.927	.021
Age^b^	0.03	0.00	0.025	0.031	1.03	1.025	1.031	<.001
Ethnicity								
Māori	−1.24	0.07	−1.386	−1.099	0.29	0.250	0.333	<.001
Pacific peoples	−0.22	0.11	−0.427	−0.010	0.80	0.652	0.990	.040
Asian	0.51	0.08	0.346	0.666	1.66	1.414	1.946	<.001
Other	0.65	0.25	0.168	1.129	1.91	1.182	3.092	.008
Conservatism	0.36	0.02	0.330	0.394	1.44	1.391	1.482	<.001
System Justification	−0.13	0.02	−0.165	−0.092	0.88	0.848	0.912	<.001
SDO	0.91	0.02	0.868	0.954	2.49	2.382	2.596	<.001
RWA	0.18	0.02	0.148	0.220	1.20	1.159	1.247	<001

Abbreviation: OR, odds ratio.

^a^ Man is the comparison gender group for each of the listed groups.

^b^ Age at Time 1 (2009). New Zealand European/Pākehā is the comparison ethnic group for each of the listed groups. *N* = 42,977.

Beginning with the demographic predictors, the odds of belonging to the *Reactionaries* (vs. *Progressives*) were about half lower for women (OR = 0.56, *p* < .001) and people who identified as gender‐diverse (OR = .61, *p* = .021) relative to men and only slightly higher for older (vs. younger) people (OR = 1.03). Turning to ethnicity, the odds of belonging to the *Reactionaries* (vs. *Progressives*) were about 71% lower for Māori relative to Pākehā (OR = 0.29), and about 20% lower for Pacific peoples relative to Pākehā (OR = 0.80, *p* = .040). Conversely, the odds of belonging to the *Reactionaries* (vs. *Progressives*) were about 66% higher for Asian people than Pākehā (OR = 1.66). Although participants in the ‘Other’ group had 191% higher odds than Pākehā of belonging to the *Reactionaries* (vs. *Progressives*; OR = 3.11, *p* = .008), these results should be treated with caution given their small sample size.

Moving to the socio‐political predictors, the odds of belonging to the *Reactionaries* (vs. *Progressives*) were ~ 44% higher for individuals high (vs. low) in conservatism (OR = 1.44), 249% higher for individuals high (vs. low) in SDO (OR = 2.49), and 20% higher for individuals high (vs. low) in RWA (OR = 1.20). Unexpectedly, the odds of belonging to the *Reactionaries* (vs. *Progressives*) were about 9% lower for individuals high (vs. low) in system justification (OR = 0.88).

### Political party support

**FIGURE 2:**
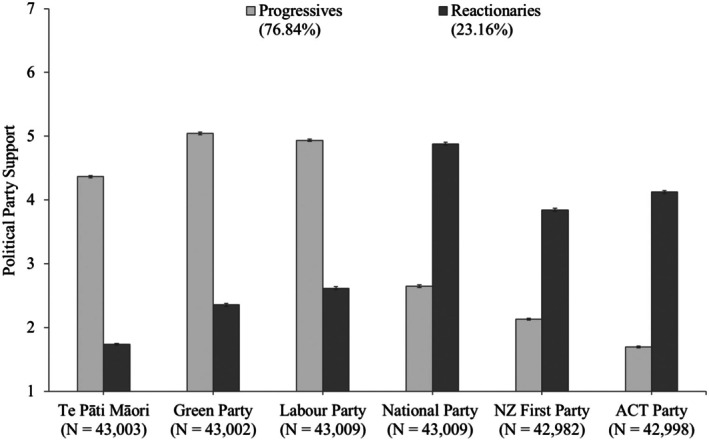
Mean support for Aotearoa's primary political parties across classes.

We next examined support for the primary political parties in Aotearoa across the two classes using a distal three‐step approach wherein class membership was used to predict party support. To determine whether the classes differed significantly in party support, we then conducted equality tests of the means. Figure 2 shows the two classes differed significantly in their support for each of the focal political parties. Indeed, the *Progressives* expressed more support than the *Reactionaries* for the Labour party (*ꭓ* ^2^_(1)_ = 21,799.62, *p* < .001), Green Party (*ꭓ* ^2^_(1)_ = 33,890.84, *p* < .001) and Te Pāti Maōri (*ꭓ* ^2^_(1)_ = 49,475.10, *p* < .001). In contrast, the *Reactionaries* expressed more support than the *Progressives* for National (*ꭓ* ^2^_(1)_ = 21,325.00, *p* < .001), New Zealand First (*ꭓ* ^2^_(1)_ = 13,661.21, *p* < .001) and ACT (*ꭓ* ^2^_(1)_ = 30,230.00, *p* < .001). Thus, the *Progressives* were more supportive of left‐wing parties, while the *Reactionaries* were more supportive of right‐wing parties.

## DISCUSSION

Consistent with global trends, Aotearoa is seeing a surge of passionate support for *and* against Indigenous rights in mainstream politics (for a review, see Corlett, 2024a) and public protest (e.g. Corlett, 2024b; Macdonald, 2024). Yet, no research to date has explored polarization in attitudes towards Indigenous rights in the public at large (i.e. outside of party voter comparisons; Satherley et al., 2020), nor during the height of this clash. Here, we address these gaps by estimating distinct trajectories of change in two ideologies that justify and promote colonial inequities (Bertenshaw, Sibley, & Osborne, 2025; Greaves et al., 2014; Osborne et al., 2017; Sibley & Osborne, 2016) from 2009 to 2025. Given the divisive political climate, we theorized that historical negation and symbolic exclusion polarized over time.

### Ideological polarization

As expected, results revealed two opposing trajectories of change in the Dark Duo. Specifically, we identified a dominant subgroup (76.84%) of *Progressives* with lower mean levels of historical negation and symbolic exclusion that gradually declined over time. Conversely, a smaller subgroup (23.16%) of *Reactionaries* had higher mean levels of the Dark Duo that increased across the assessment period. Accordingly, our findings indicate bicultural and anti‐bicultural ideological positions increasingly diverged over time, reflecting a process of polarization in the Dark Duo.

Although the distinct trajectories reflected only modest change in opposing directions, ideologies are conceived as relatively stable belief systems that only gradually shift over time (Jost, 2006; Page & Shapiro, 2010). Thus, the change observed here is notable, and the movement from parallel trajectories to significantly diverging paths captures a process of polarization in beliefs about Indigenous rights that warrants close attention. Indeed, the quadratic components in these opposing change trajectories suggest that polarization in colonial ideologies and the social and political consequences *may* become increasingly pertinent in the coming years. Thus, it is crucial to continue monitoring these groups and consider potential interventions.

The accelerated rates of change observed in the second half of the 2010s corresponds with the heightened salience of Indigenous rights in Aotearoa, with the implementation of numerous equity‐based bicultural policies under the Labour government. They also coincide with broader international changes to racial discourse amplified by Donald Trump's racially charged rhetoric. As the political sphere grew increasingly divided over Indigenous rights and racial justice through the 2020s, so, too, did colonial ideologies within the public. Thus, ideological polarization appears to intensify during heightened political action and discourse, with stronger political positions deepening division rather than uniformly shifting the public (Hetherington & Weiler, 2009). Our results thus help identify when and how ideologies polarize and contribute to debates on political and ideological change in the public (e.g. Abramowitz & Saunders, 2008; Fiorina & Abrams, 2012). With the global rise of far‐right, nationalist, and racist politics, understanding these patterns is increasingly important.

### Rise of reactionary change

The polarization process began to accelerate in the mid‐2010s when the *Reactionaries* started to increasingly endorse the Dark Duo. These results corroborate theories linking political polarization to the rise of reactionary movements (Norris & Inglehart, 2019). Certainly, the progressive ‘woke’ ideals that came to define the 21st century were targeted by the recent right‐wing swing, reflecting reactionary backlash to advances in social justice and their growing mainstream acceptance. In Aotearoa, the wave of bicultural policies passed under Labour was met with far‐right protests, including the ‘Stop Co‐Governance’ tour (Macdonald, 2024). Crucially, this small (yet vocal) anti‐bicultural movement expanded into the mainstream through the racially charged agendas of the current ruling parties: National, ACT and New Zealand First (see Corlett, 2024a; Eketone, 2024). Accordingly, social media saw a rise in anti‐Māori and white supremacist discourse (The Disinformation Project, 2023). The amplification of anti‐Māori attitudes, reinforced by the normalization of racial prejudice in politics (Crandall et al., 2018), likely explains the rise in anti‐bicultural ideologies among *Reactionaries*.

Consistent with this interpretation, the most pronounced shift we identified was the *Reactionaries'* steep incline in symbolic exclusion. Denying symbolic equality is considered more overtly prejudiced and less socially acceptable than opposing reparations, which remain controversial. In fact, settler colonizers aiming to maintain inequality sometimes embrace the Indigenous culture to create an image of biculturalism and distract from material inequities (Bertenshaw, Houkamau, et al., 2025; Rivera Pichardo et al., 2023). Although the *Reactionaries'* slower increase in historical negation may be (partly) due to a ceiling effect, the rapid rise in symbolic exclusion indicates a shift away from more subtle methods of maintaining material inequality towards more openly prejudiced attitudes. These results align with theories suggesting that racist discourse in mainstream politics helps normalize overtly prejudiced views across the broader public (Arnold et al., 2025; Crandall et al., 2018; The Disinformation Project, 2023).

Importantly, the *Reactionaries* exhibited accelerating inclines in historical negation and (even more so) symbolic exclusion, highlighting their increasingly unrestrained opposition to colonial equality. As such, results affirm the dangerous path of this subgroup and the need to intervene for Indigenous rights. Additionally, these findings support the need to counter explicit anti‐Indigenous beliefs, alongside the tokenistic approaches that received more attention in recent years (Bertenshaw, Houkamau, et al., 2025; Dovidio et al., 2017). It is also important to note that the reactionary subgroup is substantially smaller (23.16%) than the progressive subgroup. Thus, our prevalence estimates suggest that the rise in reactionary beliefs reflects the views of only a minority of adults in Aotearoa, signalling an inconsistency between the current anti‐Māori political agenda and the preferences of most New Zealanders.

### Prevalence of progressive change

Our model estimates that most New Zealanders (76.84%) are oriented towards a more progressive future that recognizes the responsibility of contemporary Aotearoa to address colonial injustices and the belonging of Māori culture to the nation's core institutions. This ideological path is consistent with the advancement of bicultural policies and discourse under the Labour government (New Zealand Parliament, 2022a, 2022b) and the widespread critique of renewed racism following the BLM movement. The recent low levels of the Dark Duo coincide with powerful public pushback against anti‐Māori attitudes in mainstream politics (see Corlett, 2024b). Thus, the current study corroborates mean‐level declines in the Dark Duo among settler colonizers from 2009 to 2018 (Bertenshaw et al., 2026) and extends this pattern to most adults in Aotearoa. Contrary to the current government's anti‐Māori agenda, results suggest that the dominant ideological path of adults in Aotearoa is best represented by policies that challenge material and representational inequities between Pākehā and Māori. Although the *Progressives'* declines in symbolic exclusion decelerated over time (likely reflecting a floor effect), their declines in historical negation accelerated, suggesting their progressive path may continue in the coming years.

We should note that further attitudinal growth is required to challenge colonial inequities between *both* subgroups. Firstly, historical negation is still considerably endorsed among the *Progressives*. The relative agreement with this ideology across classes illustrates that denying the ongoing harm of colonial injustices is normalized. Because the recognition of reparative responsibilities threatens the material privilege of the colonizer group, it is often side‐stepped or minimized even among ‘progressives’ (Rivera Pichardo et al., 2023). Other beliefs also reenact colonial harms and reinforce colonial hierarchies among seemingly progressive individuals. For example, beliefs of Western superiority are normative and structurally ingrained (for a review, see Rivera Pichardo & Bertenshaw, n.d.), and progressive support for Indigenous rights often stops at bicultural equality *within* a settler colonial structure, falling short of revolutionary decolonial changes, including self‐determination (Simon, 2022, 2023). Thus, the dominant path of progress in Aotearoa may be limited and represents only one element of a broader path to equality. Collectively, our results support the malleability of specific expressions of colonial biases and objectives (such as the Dark Duo), which helps explain the varied support for Indigenous rights, while reiterating the enduring nature of the holistic colonial mindset.

### Demographic and socio‐political predictors

Reinforcing past work showing higher support for Indigenous rights among Indigenous relative to colonizer groups (Bergmann, 2024), our results indicate that Māori and Pacific peoples had stronger tendencies for progressive change than Pākehā. Pākehā were, however, more likely than Asian participants to be on the progressive rather than reactionary trajectory. The literature on inter‐minority relations identifies both coalitional attitudes between structurally disadvantaged groups, based on perceived commonality, and intergroup derogation, driven by social identity threat (Craig & Richeson, 2012; Sanchez, 2008). When groups perceive their experiences of discrimination as distinct or competing, this can foster outgroup derogation (Craig & Richeson, 2016). In Aotearoa, discrimination towards Asian communities and Māori differs substantially in its foundations (e.g. anti‐immigrant sentiment vs. colonial logics) and common expressions, such as narratives of unfair fortune among Asian peoples (e.g. stereotypes blaming Chinese buyers for the housing crisis) and fair misfortune among Māori (e.g. stereotypes of Māori as lazy). Additionally, biculturalism is often positioned against multiculturalism in social discourse, fuelling myths that Māori rights impede broader racial equality (see Ward & Liu, 2012). Together, these dynamics may lead Asian communities to perceive symbolic and material threats to their position in society, fostering historical negation and symbolic exclusion.

Age and gender also emerged as key predictors of ideological trajectories. Corroborating patterns of more progressive views among women (Cornelis et al., 2009; Villano & Zani, 2007; Zubielevitch et al., 2023), gender‐diverse individuals (Albaugh et al., 2025) and younger people (Dozo, 2015; Huang et al., 2018; Sidanius & Pratto, 1999), results suggest these groups are more likely to exhibit progressive ideological shifts over time. Thus, men and older individuals may be more susceptible than their counterparts to a reactionary movement against Indigenous empowerment. By identifying Pākehā, Asian individuals, men and older people as most prone to reactionary mobilization, these results imply that reactionary change is (mostly) concentrated among structurally advantaged groups, with other sources of privilege aligning to defend colonial hierarchies. Our results also identify key populations for targeted interventions to reduce anti‐Indigenous mobilization.

As expected, conservatism, SDO and RWA predicted membership in the *Reactionary* subgroup. These associations reinforce the theorized link between general orientations towards inequality and ideologies that specifically justify colonial inequities (e.g. Satherley & Sibley, 2018; Sibley & Osborne, 2016). In doing so, results corroborate the anti‐egalitarian motives of the reactionary trajectory and expand understanding of why this movement emerges. For example, the association between *Reactionaries* and conservatism is consistent with the effects of rising and intensifying right‐wing politics (Arnold et al., 2026; Crandall et al., 2018). Conversely, links with SDO and RWA suggest that threat‐based and competitive motives underlie anti‐Indigenous movements and corroborate theories that differences in authoritarianism foster polarization (Hetherington & Weiler, 2009). Although our findings do not speak to causality, past research demonstrates that increases in SDO and RWA precede increases in the Dark Duo (Osborne et al., 2021). Thus, challenging these general preferences for hierarchy may help attenuate the reactionary, anti‐Indigenous movement.

Although the associations conservatism, SDO and RWA had with membership in the *Reactionaries* subgroup was expected, the odds of being in the *Reactionaries* subgroup unexpectedly decreased as a function of system justification. Considering their growing endorsement of anti‐bicultural ideologies and alignment with other anti‐egalitarian attitudes, the *Reactionaries'* dissatisfaction with the system implies they may be seeking *more* than the current level of inequality through less biculturalism. This finding corroborates research revealing dissatisfaction with the system among reactionary movements (Caricati & Rossi, 2024; Liekefett & Becker, 2022; Lilly et al., 2025). Alongside more openly racist rationales (e.g. outright denial of Indigenous rights), one common, insidious framing that may foster this perspective is the ‘myth of Māori privilege’ (see Meihana, 2023). With this, equity‐based policy is framed as promoting inequality due to ‘unequal treatment’, without recognition of the unequal starting point and systemic biases. The greater discontent with the current system among the Reactionaries reinforces our understanding of this subgroup as reactionary (i.e. actively pushing for change in the opposite direction) rather than simply aiming to preserve existing traditions and norms (Thomas & Osborne, 2022).

### Political preferences

The two opposing classes of change also demonstrated opposing support for the mainstream political parties in Aotearoa. Specifically, the *Progressives* expressed more support for the left‐of‐centre Labour Party, Green Party, and Te Pāti Māori—parties that endorse bicultural policies and, in the case of the Green Party and Te Pāti Māori, decolonial orientations. In contrast, the *Reactionaries* expressed the most support for the right‐wing National, ACT, and New Zealand First parties, which pushed to restrict the rights of the Indigenous community (Corlett, 2024a; Eketone, 2024). As such, the party preferences of the *Reactionaries* correspond with their increasingly strong colonial ideologies. Collectively, results corroborate the associations found between the Dark Duo and party preferences (Greaves et al., 2014; Satherley et al., 2020), and the distinct left–right divides between our classes reinforce the corresponding pro‐ and anti‐egalitarian goals of the *Progressives* and the *Reactionaries*.

Although our analysis does not provide insight into causality between party support and the trajectories of change in the Dark Duo, these political and ideological perspectives are likely to reinforce one another given their shared agendas. Certainly, scholars indicate a bidirectional association between the Dark Duo and conservative party preferences (Greaves et al., 2014) and warn us of the influence of prejudiced discourse among political elites on voters (Crandall et al., 2018; Schmidt‐Catran & Czymara, 2023). Thus, the anti‐bicultural agenda of right‐wing parties should—theoretically—reinforce colonial ideologies among their supporters, and the increasing justification of colonial inequities should reinforce support for parties opposing biculturalism. Conversely, pro‐bicultural ideologies and party attitudes should create a progressive cycle. An interplay of this sort between ideological trajectories and political party support could further propel polarization.

### Caveats and strengths

It is important to note several methodological limitations. First, the RWA scale includes double‐barrelled items, which may limit construct precision. However, the scale was reliable and retained for consistency with the literature. Second, women are over‐represented due to higher response rates, reducing sample representativeness. Third, our gender measure changed over time. Whereas early waves conflated sex and gender and excluded gender‐diverse individuals by using a binary male/female item, later waves allowed open‐ended responses. As such, we treated gender as time‐invariant and priority‐coded gender‐diverse – including individuals who changed gender across waves—to address this limitation. This approach does not, however, perfectly represent gender‐diverse individuals nor capture the nuances of gender fluidity. Finally, given the longitudinal nature of these data, systematic attrition could impact our results. Although robustness analyses revealed some systematic attrition (see Table S2), inconsistent responders and those who withdrew from the study were no more likely than consistent responders to belong to the *Reactionaries* subgroup. Thus, systematic attrition is unlikely to have impacted the change trajectories identified here.

Despite these limitations, our large, national probability sample provides a unique opportunity to examine distinct trajectories of ideological change in the public over 16 years. Due to the methodological demands, most psychological research relies on cross‐sectional comparisons (e.g. Bergmann, 2024) to examine a *state* of polarization, or repeated cross‐sectional assessments to infer change (e.g. Iyengar & Krupenkin, 2018) and focuses on polarization between existing groups (e.g. along voter lines; Satherley et al., 2020). In contrast, our sample and person‐centred analyses allow us to directly estimate a *process* of polarization in colonial ideologies over time among the general public. Furthermore, it enables us to estimate the prevalence of distinct trajectories, predictors of class membership and associated outcomes. Thus, the current study increases understanding of when, how and why ideological polarization occurs, and the diverse ways in which ideologies that uphold colonial inequities evolve over time.

We identify this process of ideological polarization in the settler colonial context of Aotearoa. Given the unique socio‐historical context of colonization that the Dark Duo speaks to, our findings cannot illuminate global patterns of polarization. They do, however, echo the polarizing politics of Indigenous, racial and social justice around the world. Considering the presence of the Dark Duo in other colonial contexts (Castro et al., 2022; Herkimer et al., 2025; Rivera Pichardo et al., 2023) and the links between these trajectories and more general preferences for social hierarchies, these shared political trends highlight the potential polarization of colonial ideologies elsewhere. Accordingly, our results encourage investigation of distinct trajectories of colonial ideologies across various colonial contexts to develop a broader understanding of global polarization towards Indigenous rights.

## CONCLUSION

Our results corroborate national and global patterns of political polarization around Indigenous rights by demonstrating a process of polarization in colonial ideologies through the 2010s and 2020s. Whereas most adults exhibited gradual declines in the Dark Duo, a smaller subgroup displayed increasingly high historical negation and symbolic exclusion. Results thus illustrate the ideological processes underlying clashing responses to biculturalism and Indigenous rights, corroborate concerns of a reactionary movement against Indigenous empowerment, and reveal who is more susceptible to reactionary mobilization. Notably, our prevalence estimates indicate that the rise in anti‐bicultural attitudes represents only a small segment of adults in Aotearoa. Instead, the current study suggests that the ideological trajectory of most adults in Aotearoa would be better represented by a shift towards the inclusion of Indigenous culture in mainstream systems and recognition of contemporary responsibilities to address colonial injustices.

## AUTHOR CONTRIBUTIONS

**Chris G. Sibley:** Conceptualization; funding acquisition; writing – review and editing; supervision; data curation; investigation. **Danny Osborne:** Conceptualization; writing – review and editing; supervision; investigation. **Zoe Bertenshaw:** Conceptualization; funding acquisition; writing – original draft; visualization; writing – review and editing; formal analysis; investigation.

## FUNDING INFORMATION

Data collected for the current study (and larger New Zealand Attitudes and Values Study) was funded by a grant from the Templeton Religion Trust [TRT‐2021‐10418]. Additionally, this research was funded by a University of Auckland Doctoral Scholarship awarded to Zoe Bertenshaw.

## CONFLICT OF INTEREST STATEMENT

None declared.

## ETHICS STATEMENT

The New Zealand Attitudes and Values Study was approved by the University of Auckland Human Participants Ethics Committee on 26/05/2021 for 6 years until 26/05/2027, Reference Number UAHPEC22576.

## INFORMED CONSENT

All participants gave their informed consent prior to their inclusion in the study, and responses were anonymized and confidential (for more information, see https://osf.io/75snb/).

## Supporting information


Tables S1–S2

## Data Availability

Our data are part of the New Zealand Attitudes and Values Study (NZAVS). Full copies of the NZAVS data files are held by all members of the NZAVS management team and advisory board. A de‐identified dataset containing the variables analysed here is available upon request from the corresponding author or any NZAVS advisory board member for the purpose of replicating or checking our results. The complete NZAVS survey and the *Mplus* syntax to run these analyses are available on the OSF: https://osf.io/r4edc/?view_only=4aa8c21167494ad9b877bf6722f5c854. We report all exclusions in the study. Our hypotheses and syntax were pre‐registered and can be found here: https://osf.io/jmw9e/?view_only=8ca944b647384fe88ef3da48e5cc453a.

## Footnotes

^1^
Consistent with decolonial practice, we use the Māori name, *Aotearoa*, throughout.

^2^
Ethnicity was unintentionally omitted as a predictor in our pre‐registration. We also included an additional wave of data that was collected during the review process.

^3^
To use gender as a predictor, we treat it as time‐invariant and priority code with gender‐diverse prioritized (over man and woman), which included individuals who changed gender across waves. As a result, we can also provide (imperfect) estimates of gender‐diverse individuals in Times 1–5 based on data from later waves.

^4^
Due to space constraints at Time 1, system justification was assessed using two items (*r =* .33, *p* < .001).

^5^
Due to space constraints at Time 2, RWA was assessed using five items.

^6^
Due to space constraints and party salience, support for ACT was not measured at Time 3.

^7^
Due to space constraints and party salience, support for New Zealand First was not measured at Times 1–3.
