# Supplementary material for: Polarizing trajectories of colonial ideologies: A latent class growth analysis of historical negation and symbolic exclusion
Source: Br J Soc Psychol. 2026 Aug 2;65(4):e70116. doi: 10.1111/bjso.70116 (PMC13430099; doi:10.1111/bjso.70116)
Supplement: Supplementary file 1 — Tables S1–S2 [file BJSO-65-0-s001.docx]

**Supplementary Materials**

**Table S1**

*Sample Characteristics Across the 16 Annual Assessments*

| **Variable** | **T1** | **T2** | **T3** | **T4** | **T5** | **T6** | **T7** | **T8** | **T9** | **T10** | **T11** | **T12** | **T13** | **T14** | **T15** | **T16** |
| --- | --- | --- | --- | --- | --- | --- | --- | --- | --- | --- | --- | --- | --- | --- | --- | --- |
| Sample size | 4,581 | 3,854 | 6,086 | 9,978 | 15,146 | 14,443 | 13,337 | 18,923 | 15,967 | 33,460 | 33,938 | 33,264 | 30,467 | 28,115 | 26,144 | 27,055 |
| Age (*SD*) | 49.41 (14.78) | 51.39 (14.66) | 51.48 (15.43) | 50.87 (14.71) | 49.34 (13.96) | 50.41 (13.96) | 51.63 (13.86) | 51.19 (13.65) | 52.29 (13.67) | 51.22 (13.35) | 53.05 (13.56) | 54.22 (13.43) | 55.49 (13.42) | 56.69 (13.37) | 57.65 (13.36) | 58.74 (13.17) |
| Gender |  |  |  |  |  |  |  |  |  |  |  |  |  |  |  |  |
| *Woman* | 61.3% | 62.2% | 62.4% | 62.5% | 62.9% | 62.8% | 62.4% | 62.6% | 62.8% | 63.0% | 63.5% | 63.4% | 63.4% | 63.0% | 63.2% | 63.2% |
| *Man* | 38.1% | 37.3% | 37.0% | 36.9% | 36.4% | 36.5% | 36.9% | 36.7% | 36.4% | 36.1% | 35.4% | 35.5% | 35.5% | 35.8% | 35.6% | 35.7% |
| *Gender-diverse* | 0.6% | 0.5% | 0.6% | 0.6% | 0.7% | 0.7% | 0.7% | 0.8% | 0.8% | 0.9% | 1.1% | 1.1% | 1.2% | 1.1% | 1.1% | 1.1% |
| Ethnicity |  |  |  |  |  |  |  |  |  |  |  |  |  |  |  |  |
| *Pākehā* | 91.9% | 92.4% | 94.2% | 92.0% | 92.9% | 93.1% | 93.6% | 93.6% | 94.2% | 94.3% | 94.8% | 94.9% | 95.2% | 95.3% | 95.4% | 95.4% |
| *Māori* | 20.0% | 18.9% | 15.0% | 17.3% | 15.6% | 15.2% | 14.9% | 13.9% | 13.4% | 11.4% | 10.9% | 10.9% | 10.7% | 10.4% | 10.5% | 10.5% |
| *Pacific peoples* | 5.0% | 5.0% | 4.3% | 5.5% | 4.6% | 4.6% | 4.5% | 4.1% | 4.1% | 3.3% | 3.2% | 3.2% | 3.2% | 3.1% | 3.2% | 3.1% |
| *Asian* | 4.9% | 4.8% | 4.7% | 5.4% | 5.0% | 5.0% | 4.9% | 5.1% | 4.7% | 4.7% | 4.5% | 4.4% | 4.2% | 4.1% | 4.1% | 4.0% |
| *Other^1^* | 0.1% | 0.0% | 0.2% | 0.3% | 0.3% | 0.3% | 0.3% | 0.4% | 0.4% | 0.5% | 0.6% | 0.6% | 0.6% | 0.6% | 0.6% | 0.6% |
| Historical Negation (*SD*) | 5.20 (1.46) | 5.21 (1.43) | 5.03 (1.50) | 4.99 (1.51) | 4.92 (1.52) | 4.89 (1.51) | 4.86 (1.54) | 4.80 (1.60) | 4.72 (1.63) | --- | --- | --- | --- | 4.49 (1.77) | 4.46 (1.81) | 4.43 (1.86) |
| Symbolic Exclusion (*SD*) | 3.17 (1.53) | 3.18 (1.54) | 2.92 (1.47) | 3.03 (1.52) | 2.86 (1.45) | 2.79 (1.46) | 2.71 (1.44) | 2.63 (1.40) | 2.57 (1.44) | --- | --- | --- | --- | 2.62 (1.64) | 2.58 (1.62) | 2.54 (1.62) |
| Conservatism (*SD*) | 3.75 (1.23) | 3.95 (1.29) | 3.73 (1.38) | 3.64 (1.28) | 3.62 (1.29) | 3.58 (1.31) | 3.60 (1.31) | 3.61 (1.37) | 3.57 (1.39) | 3.57 (1.38) | 3.52 (1.40) | 3.42 (1.36) | 3.48 (1.35) | 3.55 (1.40) | 3.52 (1.41) | 3.54 (1.41) |
| SJ (*SD*) | 4.50 (1.12) | 4.53 (0.99) | 4.44 (1.05) | 4.33 (1.07) | 4.46 (1.10) | 4.46 (1.15) | 4.52 (1.20) | 4.53 (1.15) | 4.46 (1.08) | 4.43 (1.05) | 4.38 (1.06) | 4.43 (1.07) | 4.26 (1.09) | 4.30 (1.07) | 4.17 (1.17) | 4.16 (1.21) |
| SDO (*SD*) | 2.57 (0.96) | 2.53 (0.93) | 2.44 (0.96) | 2.42 (0.94) | 2.32 (0.87) | 2.35 (0.90) | 2.44 (0.95) | 2.38 (0.96) | 2.35 (0.97) | 2.29 (0.95) | 2.22 (0.95) | 2.19 (0.95) | 2.21 (0.95) | 2.24 (0.97) | 2.23 (0.97) | 2.25 (0.94) |
| RWA (*SD*) | 3.55 (1.18) | 3.44 (1.20) | 3.26 (1.16) | 3.29 (1.15) | 3.30 (1.12) | 3.21 (1.13) | 3.19 (1.16) | 3.15 (1.15) | 3.08 (1.16) | 3.22 (1.14) | 3.16 (1.13) | 3.26 (1.10) | 3.33 (1.06) | 3.31 (1.07) | 3.23 (1.10) | 3.22 (1.13) |

*Note*. ^1^Other includes all other ethnic identities. SJ=system justification. SDO=social dominance orientation. RWA=right-wing authoritarianism.

**Table S2**.

*Sensitivity Analyses for the Potential Impact of Systematic Attrition on Class Membership*.

|  | Consistent Responder (*N =* 16,027) | |  | Withdrew / Inconsistent (*N* = 26,993) | |  | Mean Difference | | | |
| --- | --- | --- | --- | --- | --- | --- | --- | --- | --- | --- |
|  | *M* | (*SD*) |  | *M* | (*SD*) |  | *M* | *t* | *df* | *p* |
| Historical Negation_T1_ | 5.17 | (1.44) |  | 5.21 | (1.47) |  | 0.04 | 0.73 | 4569 | .464 |
| Historical Negation_T2_ | 5.18 | (1.39) |  | 5.21 | (1.44) |  | 0.03 | 0.51 | 3846 | .069 |
| Historical Negation_T3_ | **4.94** | **(1.51)** |  | **5.06** | **(1.49)** |  | **0.12** | **2.73** | **6078** | **.006** |
| Historical Negation_T4_ | **4.92** | **(1.54)** |  | **5.01** | **(1.50)** |  | **0.10** | **2.60** | **8904** | **.009** |
| Historical Negation_T5_ | **4.86** | **(1.54)** |  | **4.95** | **(1.51)** |  | **0.09** | **3.10** | **14665** | **.002** |
| Historical Negation_T6_ | **4.83** | **(1.53)** |  | **4.91** | **(1.50)** |  | **0.08** | **2.81** | **13967** | **.005** |
| Historical Negation_T7_ | **4.81** | **(1.57)** |  | **4.88** | **(1.52)** |  | **0.07** | **2.38** | **13313** | **.017** |
| Historical Negation_T8_ | 4.78 | (1.63) |  | 4.81 | (1.58) |  | 0.04 | 1.40 | 18905 | .163 |
| Historical Negation_T9_ | 4.69 | (1.65) |  | 4.73 | (1.61) |  | 0.04 | 1.43 | 15945 | .152 |
| Historical Negation_T14_ | **4.46** | **(1.78)** |  | **4.53** | **(1.74)** |  | **0.06** | **3.00** | **27618** | **.003** |
| Historical Negation_T15_ | 4.47 | (1.81) |  | 4.43 | (1.80) |  | −0.04 | −1.82 | 25912 | .069 |
| Historical Negation_T16_ | 4.43 | (1.87) |  | 4.43 | (1.84) |  | 0.00 | 0.05 | 26580 | .958 |
| Symbolic Exclusion_T1_ | **3.02** | **(1.45)** |  | **3.21** | **(1.54)** |  | **0.18** | **3.14** | **4567** | **.002** |
| Symbolic Exclusion_T2_ | 3.11 | (1.50) |  | 3.20 | (1.55) |  | 0.09 | 1.51 | 3842 | .132 |
| Symbolic Exclusion_T3_ | **2.86** | **(1.48)** |  | **2.95** | **(1.47)** |  | **0.08** | **1.96** | **6080** | **.050** |
| Symbolic Exclusion_T4_ | 2.98 | (1.52) |  | 3.04 | (1.52) |  | 0.07 | 1.75 | 8918 | .081 |
| Symbolic Exclusion_T5_ | **2.78** | **(1.42)** |  | **2.89** | **(1.46)** |  | **0.11** | **3.93** | **14692** | **< .001** |
| Symbolic Exclusion_T6_ | **2.72** | **(1.46)** |  | **2.81** | **(1.46)** |  | **0.10** | **3.49** | **14394** | **< .001** |
| Symbolic Exclusion_T7_ | **2.63** | **(1.42)** |  | **2.75** | **(1.44)** |  | **0.12** | **4.47** | **13328** | **< .001** |
| Symbolic Exclusion_T8_ | **2.59** | **(1.41)** |  | **2.64** | **(1.40)** |  | **0.05** | **2.11** | **18909** | **.035** |
| Symbolic Exclusion_T9_ | 2.55 | (1.45) |  | 2.59 | (1.44) |  | 0.04 | 1.59 | 15949 | .111 |
| Symbolic Exclusion_T14_ | **2.60** | **(1.64)** |  | **2.66** | **(1.63)** |  | **0.06** | **3.02** | **27621** | **.003** |
| Symbolic Exclusion_T15_ | 2.60 | (1.64) |  | 2.56 | (1.58) |  | −0.04 | −1.88 | 25904 | .060 |
| Symbolic Exclusion_T16_ | 2.55 | (1.64) |  | 2.53 | (1.58) |  | −0.02 | −0.86 | 26683 | .393 |
| Conservatism | **3.49** | **(1.40)** |  | **3.57** | **(1.35)** |  | **0.08** | **5.55** | **42975** | **< .001** |
| System Justification | 4.39 | (1.09) |  | 4.38 | (1.10) |  | −0.01 | −1.36 | 43018 | .175 |
| SDO | **2.25** | **(0.97)** |  | **2.34** | **(0.95)** |  | **0.09** | **9.12** | **43018** | **< .001** |
| RWA | **3.13** | **(1.14)** |  | **3.26** | **(1.16)** |  | **0.13** | **11.41** | **43018** | **< .001** |
| Reactionaries | 0.23 | (0.42) |  | 0.23 | (0.42) |  | −0.00 | -0.87 | 43018 | .386 |

**Robustness Analyses**

Recent evidence reveals that there is a growing sense of distrust in science amongst conservatives (Hartman et al., 2017; Kerr & Wilson, 2021). As such, systematic attrition may have impacted our results via conservative participants dropping out of the study at later assessment waves. To test this alternative explanation for our latent class growth curves, we split our sample into participants who joined and consistently stayed in the study (consistent responders; *N =* 16,027) and those who responded inconsistently over time and/or withdrew from the study (inconsistent responders; *N =* 26,993). The series of *t*-tests displayed in Table S2 reveal that inconsistent responders were significantly higher than consistent responders on historical negation at 6 of the 12 of the assessments. Inconsistent responders were also higher than consistent responders on 7 of the 12 assessments of symbolic exclusion, as well as their first assessments of conservatism, SDO, and RWA. Inconsistent responders were, however, no more likely than the consistent responders to belong to the class of *Reactionaries* (*p* = .386). Thus, although there is some evidence that inconsistent responders were slightly more conservative than their counterparts who were consistent responders, there is no reason to believe that this selective attrition impacted class membership.

**References**

Hartman, R. O., Dieckmann, N. F., Sprenger, A. M., Stastny, B. J., & DeMarree, K. G. (2017). Modeling attitudes toward science: Development and validation of the credibility of science scale. *Basic and Applied Social Psychology*, *39*(6), 358–371. <https://doi.org/10.1080/01973533.2017.1372284>

Kerr, J. R., & Wilson, M. S. (2021). Right-wing authoritarianism and social dominance orientation predict rejection of science and scientists. *Group Processes & Intergroup Relations*, *24*(4), 550–567. <https://doi.org/10.1177/1368430221992126>
